# Predictive Models for Weekly Cattle Mortality after Arrival at a Feeding Location Using Records, Weather, and Transport Data at Time of Purchase

**DOI:** 10.3390/pathogens11040473

**Published:** 2022-04-15

**Authors:** Lauren Wisnieski, David E. Amrine, David G. Renter

**Affiliations:** 1 Center for Animal and Human Health in Appalachia, College of Veterinary Medicine, Lincoln Memorial University, Harrogate, TN 37752, USA; 2 Center for Outcomes Research and Epidemiology, College of Veterinary Medicine, Kansas State University, Manhattan, KS 66502, USA; damrine@vet.k-state.edu (D.E.A.); drenter@vet.k-state.edu (D.G.R.); 3 Beef Cattle Institute, Kansas State University, Manhattan, KS 66502, USA

**Keywords:** predictive modeling, feedlot health, random forests, mortality, beef cattle

## Abstract

Feedlot mortality negatively affects animal welfare and profitability. To the best of our knowledge, there are no publications on predictive models for weekly all-cause mortality in feedlot cattle. In this study, random forest models to predict weekly mortality for cattle purchase groups (*n* = 14,217 purchase groups; 860,545 animals) from arrival at the feeding location (Day 1) to Day 42 and cumulative mortality from Day 43 until slaughter were built using records, weather, and transport data available at the time of purchase. Models were evaluated by calculating the root mean squared error (RMSE) and accuracy (as defined as the percent of purchase groups that had predictions within 0.25% and 0.50% of actual mortality). The models had high accuracy (>90%), but the RMSE estimates were high (range = 1.0% to 4.1%). The best predictors were maximum temperature and purchase weight, although this varied by week. The models performed well among purchase groups with low weekly mortality but performed poorly in high mortality purchase groups. Although high mortality purchase groups were not accurately predicted utilizing the models in this study, the models may potentially have utility as a screening tool for very low mortality purchase groups after arrival. Future studies should consider building iterative models that utilize the strongest predictors identified in this study.

## 1. Introduction

From 1994 to 2011, feedlot mortality has remained steady at around 1% [1]. Feedlot mortality has negative effects, including decreased animal welfare and reduced profitability [2]. For each percentage increase in mortality, added costs due to lower average daily gain and feed conversion ratio are estimated to amount to 1 USD/head [3]. A potential way to lower beef cattle mortality is to make management changes that may improve animal health, including the adjustment of processing procedures, preconditioning cattle, and improving the monitoring of high-risk groups of cattle [4,5]. One tool that can be used to improve the identification of cattle at-risk of mortality is predictive modeling, which is a statistical modeling method that can be used to predict future events [6]. Previous studies focused on building predictive models for bovine respiratory disease (BRD) morbidity [7,8], the number of days on feed (DOF) to first treatment [9], and identifying cattle that did not finish the production cycle normally after BRD treatment [10]. However, to the best of our knowledge, no previous studies developed predictive models for all-cause mortality in beef cattle. 

Ideally, cattle mortality can be predicted as early as possible so that interventions can be promptly implemented. Previously, we showed that weather parameters and demographic factors measured on the day of purchase are associated with cumulative BRD mortality in the first 60 days on feed [11]. In addition, Amrine et al. 2019 [8] built predictive models for the BRD risk category and included multiple predictors, including weather variables pre- and post-arrival at a feeding location, and found that models that included information from the sale barn had similar accuracy compared to models built with lot arrival data. However, it is unknown if purchase data can be used to predict weekly all-cause mortality. Accurately predicting which groups of cattle are at risk of high mortality as early as the day of purchase may have benefits over only using information available when cattle arrive at the feeding location. Predictions of mortality risk generated at purchase can guide transportation and processing procedures, allowing for earlier intervention in groups of cattle that are expected to have high mortality shortly after arrival at a feeding location. For example, antimicrobials can be better utilized to target groups of animals with high-risk animals after arrival at the feedlot [4].

The objective of this study was to build a series of random forest models to predict weekly mortality from arrival at a feeding location (Day 1) to 42 days on feed (DOF) (Day 42) and cumulative mortality from Day 43 to slaughter in purchase groups from one large commercial beef cattle feeding system based on records, weather, and transport data available at the time of purchase.

## 2. Results

### 2.1. Descriptive Statistics

Descriptive statistics for the full sample are presented in Table 1 and Table 2. The training dataset contained 9967 purchase groups and the test dataset contained 4250 purchase groups. Ninety-eight percent of deaths were attributed to BRD. Most of the purchase groups were all steers, contained all calves or both calves and yearlings (versus all yearlings), and had weaned animals. The majority of purchase groups were purchased in winter, were purchased from the Southern United States (US) or were from mixed sources, and were purchased from the auction or had a mix of auction/contracted animals.

### 2.2. Model Results 

The optimal random forest mtry parameters (number of randomly selected predictors to choose from at each split in the trees) identified from the grid search were 2 (Weeks 1–4 and Week 6) and 3 (Week 5 and Day 43 to slaughter). The optimal number of trees (which are pooled to form a forest of which predictions are made) for the models were 1000, 1000, 1300, 1300, 1300, 1500, and 1000 for Weeks 1–6 and for Day 43 to slaughter, respectively. The mean and median DOF was 100.5 days and 82 days, respectively. The final models included 17 variables, as described in Table 3. 

Overall weekly model results are presented in Table 4. The accuracy of the models was highest for Week 1 and decreased as weeks increased. Accuracy was very high for Week 1–Week 6 (>90%) but was much lower for Day 43 to slaughter (<70%). Overall, the most important predictor variables were maximum temperature and purchase weight.

Table 5 and Table 6 describe average model performance (RMSE and accuracy) when stratified by different purchase group characteristics. In general, the models performed worse in high mortality purchase groups (>2% mortality) and in purchase groups with high-risk characteristics. For example, the models performed worse in purchase groups with calves and mixed groups compared to purchase groups with only yearlings. Model accuracy (the ability of the model to predict mortality within 0.25% of actual mortality) was low for purchase groups with high weekly mortality (Table 6). Model performance, as measured by root mean squared error (RMSE), among high mortality purchase groups (>5%) was best in the Week 4 and Week 5 models and worst in the Week 1 model. However, even in the best performing weeks for high mortality purchase groups, the RMSE estimates were high (0.083 and 0.081 for Week 4 and Week 5, respectively). RMSE estimates can be interpreted on the scale of outcome (% mortality) by multiplying by 100. Therefore, an RMSE of 0.083 indicates that the predicted mortality deviated, on average, 8.3% from observed mortality. The models had high accuracy and low RMSE estimates for purchase groups with low weekly mortality (0% to ≤2%).

In sensitivity and specificity analyses to assess the model’s ability to accurately detect purchase groups with low mortality (0% to 0.25% or 0% to 0.50%), Weeks 3–5 had the best balance of sensitivity and specificity, although specificity was low (Table 7). The Week 1 model had low specificity because the models performed poorly at identifying the few purchase groups that had higher mortality. The Day 43 to Slaughter model had 100% specificity because there were no purchase groups with low mortality, which the models accurately predicted.

## 3. Discussion

Predicting feedlot mortality risk at the time of purchase could help determine when changes in processing or management are needed for certain purchase groups. For example, purchase groups that are high-risk may be sent to a backgrounding facility or may have modified processing procedures that can reduce stress [5]. Intervening right after purchase is ideal since it is one of the earliest points for intervention. Likewise, identifying purchase groups that are low-risk could be beneficial for personnel management as-well-as judicial antimicrobial administration. Therefore, our objective was to build all-cause mortality predictive models using data available at the time of purchase. 

Random forest models for weekly mortality of beef cattle after arrival at a feeding location were built using cattle records, weather information, and transport data available at the time of purchase. Even though the models had high accuracy (>90%), the RSMEs were large when considering the scale of weekly percent mortality at a feedlot. In order to illustrate this, mortality in the first 30 days of the feeding period averaged 0.40% and 0.35% in heifers and steers, respectively, in a study by Vogel et al. 2015 [12]. Therefore, an RMSE of approximately 1%, as reported in some of our models, would be a large margin around the actual percent mortality. The models performed more poorly among purchase groups with high weekly mortality. There are several possible reasons for this finding. One major reason is that predictions generated from predictive models tend to favor the majority class, and predictions are shrunk towards the sample mean [13]. Since the average overall weekly mortality was close to 0%, the predictions were shrunk towards 0%, and the models tended to under-predict for high-mortality purchase groups. In general, the models also performed worse for purchase groups with characteristics associated with a higher risk of morbidity and mortality. For example, purchase groups with known risk factors for mortality such as calves (versus yearlings), unweaned calves, lower average arrival weight, calves purchased from auctions, and calves purchased in the fall had less accurate predictions compared with their lower-risk counterparts [11,14,15,16]. This is also likely due to shrinkage towards the sample mean and consistent with under-prediction of mortality risk. 

Predictions were less accurate as the weeks progressed, likely because the predictions were farther and farther away from the data used to generate them, and there were intervening events and treatments. For example, transport, processing, co-mingling with other purchase groups, weather after arrival, and treatments all affected mortality risk and were not included in the prediction models [5,11,14]. Cattle processing is of concern because the perceived risk on arrival can influence how cattle are processed. High-risk cattle were more likely to receive metaphylaxis and potentially other preventative measures, which affects mortality risk. 

In the groups that had 0% mortality, random forest models were highly accurate (100%) with low RMSE values (range of 0.001 to 0.003) for Week 1 through Week 6. Although models were less accurate at identifying groups with greater than 0% mortality, there could be significant utility in models that accurately identify groups at low risk of mortality (0%). For example, fewer personnel can be allocated to monitoring low-risk groups. However, this will lead to an increased number of false negatives and decreased sensitivity (i.e., high-risk groups identified as low-risk). The models with the best balance of sensitivity and specificity for detecting low mortality purchase groups (0–0.50% mortality) were models for Weeks 3–5. 

Although the models performed relatively poorly on high-mortality groups, the results still have utility in that models performed well on low mortality purchase groups (0% to 0.25% mortality or 0% to 0.50% mortality), and they demonstrated the relative predictive ability of data at different time points. The strongest predictors can inform future studies aimed to build predictive models for mortality. It is interesting to note that the strongest predictors varied by week. In earlier weeks, the maximum temperature was a strong predictor, but in later weeks purchase weight became more important. Higher temperatures contribute to heat stress, which increases the risk of morbidity and mortality [11,17]. The predictive ability of the maximum temperature on the day of purchase may decrease over time since the weather conditions have likely changed after arrival at the feeding location. Purchase weight, which was consistently a strong predictor but increased in importance as weeks progressed, was also consistently associated with morbidity and mortality in multiple studies [7,11,14,16]. Age (calf and mixed purchase groups versus yearling only purchase groups) was a strong predictor in later weeks. Age is a rough proxy for body weight, but calves also may have a higher risk of morbidity and mortality after arrival at a feeding location compared to yearlings because they have not been exposed to as many pathogens over time, making them more susceptible [18,19]. The region was a strong predictor in Weeks 3–5. Studies found a significant association between the region of origin and morbidity and mortality in beef cattle [15,16,20]. This could be due to increased shipping distances from farther regions, which may increase transportation stress [21]. Of note, in the weeks with the highest sensitivity and specificity for detecting low mortality purchase groups (Weeks 3–5), the strongest predictors were purchase weight, number of cattle in the purchase group, region, and weaned status. Future studies should incorporate these predictors in models with consideration of their varying predictive ability over time. 

To the best of our knowledge, the present study is the first to attempt to build predictive models for all-cause mortality. Other studies that have built models for predicting BRD morbidity and the probability of not finishing the production cycle also had difficulty producing accurate predictions [9,22] or had very inconsistent accuracy based on factors such as DOF and arrival weight [7]. However, Kayser et al. 2019 [23] built models with high predictive accuracy for BRD incidence using feeding behaviors, so it is possible that feeding behaviors could be a useful predictor for mortality since BRD is a major cause of death in feedlot cattle [24]. Several potential predictors for BRD were discussed in a narrative review by Wisnieski et al. 2021b [25]. The review highlighted complete blood counts, acute phase protein concentrations, and data from precision livestock farming technologies as predictors that should be evaluated further for their predictive ability [25]. In addition, predictions also can be improved by building models that include more information after arrival at a feeding location and update daily or weekly with new estimates. Babcock et al. 2013 [7] found that including daily morbidity data after arrival improved morbidity predictions for cohorts of cattle that were determined to be high-risk at arrival. Similar to our findings, the authors found that the models generally performed better for low-risk cohorts. 

There are several limitations of this study. One limitation is that purchase-group level data were used throughout the analysis, but purchase groups were combined into different lots at the feeding location. However, at the time of purchase, it was unknown which purchase groups would be combined into lots, so the analysis reflected real-life management practices. This could have lowered the predictive ability of the models because lot-level factors, such as the degree of co-mingling of purchase groups into lots, can affect disease risk [26]. In addition, only one feeding operation was utilized for the analysis. The results cannot be generalized outside of the study population without further validation. However, there were several feeding locations in different states. The results of this study can be used to guide future predictive models that incorporate multiple feeding operations. 

In summary, records, weather, and transport data available at the time of purchase produced predictions for weekly mortality among purchase groups with wide error margins. The models performed poorly among high-risk purchase groups, underestimating their mortality risk, which indicates that the models would not accurately identify high-risk purchase groups for additional health interventions. However, the models performed well for low-mortality purchase groups; therefore, the models could potentially serve as a screening tool to identify low mortality purchase groups. Model accuracy decreased as weeks progressed, however, the sensitivity and specificity of the models to detect low mortality purchase groups were highest in Weeks 3–5. Future studies should consider utilizing some of the strongest predictors that were identified in this study and build daily or weekly risk models that can incorporate more information as days on feed increase. In addition, future studies can investigate the use of different modeling strategies, such as building separate models for low versus high-risk purchase groups or building predictive models to identify categories of mortality risk (quartiles or quintiles of mortality). 

## 4. Materials and Methods

Animal use approval was not needed because the data were ascertained through an existing dataset. All data management and statistical analyses were performed in Stata version 14.2 (StataCorp, College Station, TX, USA) and R version 3.6.0 (R Foundation for Statistical Computing, Vienna, Austria). 

### 4.1. Data Sources 

Information on demographics, purchase and backgrounding locations, and health outcomes were obtained through an existing operational data system from a large commercial feeding operation located in the midwestern United States. The unit of analysis was the purchase group, which was defined as animals that are purchased, grouped, and then transported together to the backgrounding or feedlot location. Prior to data cleansing, the dataset included 14,631 purchase groups. The dataset included all purchase groups from November 2015 to January 2019. More details about the feeding system are described in Wisnieski et al. 2021a [11].

Weather information only on the purchase day was used in the models. Weather information on the purchase day was downloaded using the DarkSky application program interface (API) with the “dark sky” package in R [27,28]. Daily weather data included wind bearing, apparent temperature minimum and maximum, temperature minimum and maximum, precipitation accumulation, precipitation type, ultraviolet (UV) index, dew point, humidity, and wind speed. The latitude and longitude of the purchase location and the backgrounding or feedlot location were downloaded for each purchase group through the MapQuest API with the “mqgeocode” command in Stata [29,30]. 

### 4.2. Data Partitioning

The full dataset was partitioned into test (30%) and training (70%) datasets and balanced based on quartiles of the outcome (% cumulative mortality for the entire feeding period) using the “caret” package in R [31].

### 4.3. Data Processing

Pre-processing and exploratory data analysis were completed using the training dataset. Pre-processing included visually assessing variables using histograms and box plots for unusual observations, checking for missingness, bivariable analyses with the outcome, and multicollinearity assessment [13]. The full list of variables considered for inclusion is presented in Supplementary Table S1. Variables with a large amount of missing data or were highly correlated (r > 0.90) with another variable were dropped. In total, 16 variables were dropped. Levels of categorical variables with sparse categories were combined. There were very few purchase days with precipitation; therefore, precipitation was treated as a binary categorical variable (0 = there was no precipitation, 1 = there was precipitation). Observations that were missing any of the final variables listed in Table 3 were dropped. The test data were processed the same as the training data. After processing, the final dataset included 14,217 purchase groups, consisting of 860,545 animals.

### 4.4. Random Forests

Random forests are a tree-based ensemble method that constructs a multitude of decision trees. The forest’s predictions are calculated by averaging the individual trees’ predictions. The ensemble nature of random forest models makes understanding the relationships between predictors and outcomes impossible; however, random forest models are specifically designed to optimize predictions. Random forests can be used for either classification of categorical data or for regression [32]. For this analysis, regression was used since the outcome was defined as percent mortality. The training dataset was used to build 7 random forest models: 6 models for weekly mortality from arrival at a feeding location (Day 1) to Day 42 and one model for cumulative mortality from Day 43 to slaughter, using the “caret” package in R. For each model, mortality was defined as the number that died during that time period/total number in purchase group at the time of purchase. One tuning parameter for random forests is mtry, which is the number of randomly selected variables to choose from at each split. For each model, five values of mtry between 2 and 17 were tested with approximately equal increments; then, the optimum mtry value was narrowed down to a more precise value using the “bestTune” model output [32]. For each random forest, at least 1000 trees were used. The number of trees increased from 1000 until the performance of the model leveled off [32]. RMSE values, which measure (on average) how far the residuals are from 0, were calculated for each model through five-fold cross-validation (CV) to evaluate model performance [32]. Finally, the 7 random forest models were tested for predictive ability on the test dataset. Accuracy was calculated as the percent of purchase groups that were correctly classified within 0.25% and 0.50% percent of actual weekly mortality. RMSE values and accuracy within 0.25% were reported by purchase group characteristics to assess if the models performed better in certain sub-groups of cattle. Lower RMSE values indicate that predictions have smaller deviations from the observed values. RMSE can be interpreted on the scale of mortality by multiplying RMSE values by 100. Variance importance was also reported in order to determine which variables were the strongest predictors. Variables with high importance are used more by the model to make predictions compared to variables with low importance.

In order to further examine the ability of the models to detect low mortality purchase groups, sensitivity and specificity were calculated to evaluate the ability of the models to detect purchase groups with 0% to 0.25% and 0% to 0.50% weekly mortality. Sensitivity was calculated as the percent of low mortality purchase groups that were accurately predicted to have low mortality. Specificity was calculated as the percent of purchase groups that did not have low mortality that was accurately identified as not having low mortality.

## Figures and Tables

**Table 1:** Descriptive statistics of categorical variables in purchase-group level data from a large commercial beef feeding operation in the Midwestern United States (*n* = 14,217 purchase groups).

Variable	Category	*n*(%)
Sex	Female	285 (2.0)
	Male	13,932 (98.0)
Age	Calf and mixed	11,173 (78.6)
	Yearling	3044 (21.4)
Weaned status	Unweaned and mixed	2082 (14.6)
	Weaned	12,135 (85.4)
Purchase season	Spring	4476 (31.5)
	Summer	2091 (14.7)
	Fall	2973 (20.9)
	Winter	4677 (32.9)
Geographic origin	Canada or North US	227 (1.6)
	South US or mixed	13,990 (98.4)
Source	Auction and mixed	13,588 (95.6)
	Contracted	629 (4.4)
Purchase year	2015	992 (7.0)
	2016	4441 (31.2)
	2017	5273 (37.1)
	2018	3508 (24.7)
	2019	3 (0.02)
Day of week purchased	Monday	1777 (12.5)
	Tuesday	2936 (20.7)
	Wednesday	3486 (24.5)
	Thursday	3176 (22.3)
	Friday	1962 (13.8)
	Saturday/Sunday	880 (6.2)
Week of month purchased	Days 1–7	3376 (23.8)
	Days 8–14	3624 (25.5)
	Days 15–21	3503 (24.6)
	Day 22 until EOM	3714 (26.1)
Precipitation	Yes	573 (4.0)
	No	13,644 (96.0)
Mortality	0%	4131 (29.1)
	0% to ≤2%	3007 (21.2)
	2% to <5%	3532 (24.8)
	≥5%	3547 (25.0)

**Table 2:** Descriptive statistics of numerical variables in purchase-group level data from a large commercial beef feeding operation in the Midwestern United States (*n* = 14,217 purchase groups).

Variable	Mean (SD)
Average purchase weight (kg)	307.8 (57.2)
Head (Number of cattle per purchase group)	60.6 (49.2)
Shipping distance (km)	510.5 (302.6)
Relative humidity	0.69 (0.14)
Windspeed (km/h)	10.6 (6.1)
Maximum temperature (Celsius)	15.1 (12.5)

**Table 3:** Description of predictors used in random forest models predicting all-cause mortality among purchase groups of feedlot cattle.

Predictor	Description	Coding ^1^
Age	Age of purchase group	1 = Calf and mixed, 2 = Yearling
Weaned status	Weaning status of purchase group	1 = Mixed and unweaned, 2 = Weaned
Sex	Sex of purchase group	1 = Heifer, 2 = Steer
Origin	Geographic region where purchased	1 = Canada or north US, 2 = South or mixed
Average purchase weight	Average purchase weight of purchase group	Continuous
Month	Month purchased	1 = January, 2 = February, … 12 = December
Day of week	Day of week purchased	1 = Monday, 2 = Tuesday, … 6 = Saturday/Sunday
Week of month	Week of month purchased	1 = Days 1–7, 2 = Days 8–14, 3 = 15–21, 4 = Day 22 until end of month
Season	Season when purchased	1 = Spring (March, April, May), 2 = Summer (June, July, August), 3 = Fall (September, October, November), 4 = Winter (December, January, February)
State	State where purchased, states with small sample size grouped together based on location and form regions	1 = CO, 2 = FL, GA, AL, MS, SC, 3 = ID, NV, OR, UT, CA, WY, MT, 4 = IL, OH, WI, 5 = IA, 6= KS, 7 = KY, 8 = MN, 9 = MS, AR, 10 = NE, 11 = ND, 12 = OK, TX, 13 = SD, 14 = TN, 15 = N/A (Canada)
Source	Purchase source	1 = Auction and mixed, 2 = contracted
Shipping distance	Distance from purchase location to backgrounding/feedlot location	Continuous
Head	Number of cattle in purchase group	Continuous
Relative humidity	Humidity on purchase day	Continuous
Windspeed	Windspeed on purchase day	Continuous
Maximum temperature	Maximum temperature on purchase day	Continuous
Precipitation	Precipitation on purchase day	0 = There was no precipitation, 1 = There was precipitation

^1^ Variables inputted in the final models in the format presented in this table.

**Table 4:** Description of root mean squared error (RMSE), accuracy, and variable importance of random forest models predicting all-cause mortality among purchase groups of feedlot cattle in weeks after arrival at a feeding location (*n* = 4250 purchase groups).

Model	RMSE ^1^	Accuracy within 0.25%	Accuracy within 0.5%	Most Important Variable	2nd Most Important Variable	3rd Most Important Variable
Week 1	0.009	96.16%	96.38%	Max temperature	Windspeed	Shipping distance
Week 2	0.011	93.86%	94.26%	Max temperature	Purchase weight	Season (Winter)
Week 3	0.013	92.66%	93.06%	Purchase weight	Region (Southern US)	Region (NW US)
Week 4	0.01	92.00%	92.56%	Number of cattle	Purchase weight	Windspeed
Week 5	0.009	91.46%	92.21%	Weaned status	Purchase weight	Region (Midwestern US)
Week 6	0.01	91.76%	92.14%	Purchase weight	Age	Month (September)
Day 43 to slaughter	0.041	63.51%	66.96%	Purchase weight	Max temperature	Age

^1^ RMSE reported in decimals. To convert to % mortality, multiply by 100. Max = Maximum; NW = Northwestern.

**Table 5:** Mean RMSE ^1^ of random forest models predicting all-cause mortality among purchase groups of feedlot cattle in weeks after arrival at a feeding location in test dataset stratified by purchase group characteristics (*n* = 4250 purchase groups).

Variable	Category	Week 1	Week 2	Week 3	Week 4	Week 5	Week 6	Day 43 to Slaughter	Average
Sex	Female (*n* = 82)	0.027	0.030	0.009	0.007	0.007	0.005	0.041	0.018
	Male (*n* = 4, 168)	0.008	0.010	0.013	0.010	0.009	0.010	0.041	0.014
Age	Calf and mixed (*n* = 3338)	0.010	0.011	0.013	0.010	0.010	0.011	0.044	0.016
	Yearling (*n* = 912)	0.007	0.008	0.013	0.006	0.008	0.007	0.027	0.011
Weaned status	Unweaned ^2^ (*n* = 627)	0.013	0.018	0.023	0.018	0.015	0.019	0.068	0.025
	Weaned (*n* = 3623)	0.008	0.009	0.010	0.007	0.008	0.008	0.035	0.012
Purchase weight ^3^	≤255.8 (*n* = 1067)	0.013	0.015	0.015	0.013	0.010	0.009	0.050	0.018
	255.8 to ≤302.1 (*n* = 1063)	0.004	0.010	0.011	0.008	0.008	0.013	0.043	0.014
	302.1 to ≤346.5 (*n* = 1061)	0.008	0.008	0.012	0.010	0.010	0.010	0.040	0.014
	>346.5 (*n* = 1059)	0.009	0.009	0.013	0.006	0.009	0.007	0.029	0.012
Purchase season	Spring (*n* = 1299)	0.100	0.013	0.012	0.009	0.007	0.006	0.039	0.027
	Summer (*n* = 608)	0.007	0.004	0.009	0.009	0.010	0.008	0.032	0.011
	Fall (*n* = 909)	0.012	0.013	0.015	0.012	0.013	0.016	0.044	0.018
	Winter (*n* = 1434)	0.006	0.009	0.013	0.009	0.008	0.008	0.055	0.015
Geographic origin	Canada or North US (*n* = 58)	0.002	0.002	0.006	0.003	0.005	0.003	0.027	0.007
	South US or mixed (*n* = 4192)	0.009	0.011	0.013	0.010	0.009	0.010	0.041	0.015
Source	Auction and mixed (*n* = 4061)	0.009	0.011	0.013	0.01	0.010	0.010	0.042	0.015
	Contracted (*n* = 189)	0.002	0.002	0.004	0.005	0.004	0.003	0.016	0.005
Head	≤27 (*n* = 1078)	0.015	0.017	0.021	0.014	0.015	0.017	0.070	0.024
	27 to ≤53 (*n* = 1058)	0.007	0.011	0.010	0.009	0.008	0.008	0.030	0.012
	53 to ≤77 (*n* = 1058)	0.004	0.005	0.007	0.009	0.005	0.005	0.023	0.008
	>77 (*n* = 1056)	0.004	0.004	0.007	0.005	0.004	0.005	0.021	0.007
Purchase year ^4^	2015 (*n* = 318)	0.014	0.011	0.014	0.014	0.016	0.014	0.054	0.020
	2016 (*n* = 1369)	0.009	0.011	0.013	0.011	0.010	0.012	0.047	0.016
	2017 (*n* = 1030)	0.010	0.011	0.011	0.007	0.008	0.008	0.034	0.013
	2018 (*n* = 1030)	0.004	0.010	0.014	0.009	0.008	0.008	0.037	0.013
Weekly mortality	0%	0.001	0.003	0.003	0.003	0.003	0.002	0.026	0.006
	0% to ≤2%	0.012	0.011	0.011	0.010	0.010	0.011	0.013	0.011
	2% to <5%	0.031	0.027	0.030	0.028	0.027	0.029	0.012	0.026
	≥5%	0.122	0.109	0.119	0.083	0.081	0.098	0.086	0.100

^1^ RMSE reported in decimals. To convert to % mortality, multiply by 100. ^2^ Unweaned and mixed purchase groups. ^3^ Average purchase weight (kg). ^4^ The year 2019 was omitted from table due to small sample size (*n* = 2 purchase groups).

**Table 6:** Accuracy within 0.25% of weekly mortality from random forest models predicting all-cause mortality among purchase groups of feedlot cattle in weeks after arrival at a feeding location in a test dataset stratified by purchase group characteristics (*n* = 4250 purchase groups).

Variable	Category	Week 1	Week 2	Week 3	Week 4	Week 5	Week 6	Day 43 to Slaughter	Average
Sex	Female (*n* = 82)	92.7%	89.0%	92.7%	89.0%	96.3%	93.9%	78.1%	90.2%
	Male (*n* = 4168)	96.2%	94.0%	92.7%	92.1%	91.4%	91.7%	63.2%	88.8%
Age	Calf and mixed (*n* = 3338)	96.3%	93.8%	92.0%	91.3%	90.5%	90.6%	61.9%	88.1%
	Yearling (*n* = 912)	95.6%	94.0%	95.1%	94.7%	94.9%	95.9%	69.5%	91.4%
Weaned status	Unweaned ^1^ (*n* = 627)	94.3%	91.2%	86.8%	86.3%	83.9%	85.5%	57.3%	83.6%
	Weaned (*n* = 3623)	96.5%	94.3%	93.7%	93.0%	92.8%	92.9%	64.5%	89.7%
Purchase weight ^2^	≤255.8 (*n* = 1067)	95.2%	93.1%	90.1%	89.5%	88.6%	89.6%	56.8%	86.1%
	255.8 to ≤302.1 (*n* = 1063)	96.6%	93.7%	92.9%	92.6%	91.1%	91.1%	61.4%	88.5%
	302.1 to ≤346.5 (*n* = 1061)	96.9%	94.2%	92.7%	91.0%	91.2%	90.8%	65.5%	88.9%
	>346.5 (*n* = 1059)	95.9%	94.5%	95.0%	95.0%	95.0%	95.7%	70.4%	91.6%
Purchase season	Spring (*n* = 1299)	96.9%	95.2%	94.7%	94.8%	93.8%	93.6%	62.1%	90.2%
	Summer (*n* = 608)	96.9%	95.6%	92.3%	91.1%	87.2%	90.5%	63.5%	88.2%
	Fall (*n* = 909)	94.7%	91.0%	89.6%	88.7%	88.9%	87.8%	65.6%	86.6%
	Winter (*n* = 1434)	96.1%	93.7%	93.0%	91.9%	92.8%	93.2%	63.5%	89.2%
Geographic origin	Canada or North US (*n* = 58)	98.3%	96.6%	89.7%	93.1%	93.1%	96.6%	70.7%	91.2%
	South US or mixed (*n* = 4192)	96.1%	93.8%	92.7%	92.0%	91.4%	91.7%	63.4%	88.7%
Source	Auction and mixed (*n* = 4061)	96.2%	93.8%	92.5%	91.9%	91.3%	91.7%	62.9%	88.6%
	Contracted (*n* = 189)	95.2%	95.8%	96.8%	94.7%	94.7%	94.2%	77.3%	92.7%
Head ^3^	≤27 (*n* = 1078)	98.3%	97.3%	96.0%	96.9%	95.7%	96.3%	65.8%	92.3%
	27 to ≤53 (*n* = 1058)	96.2%	94.8%	93.2%	92.2%	91.9%	92.2%	61.0%	88.8%
	53 to ≤77 (*n* = 1058)	96.0%	92.6%	92.1%	91.3%	91.5%	91.9%	64.5%	88.6%
	>77 (*n* = 1056)	94.0%	90.6%	89.3%	87.5%	87.7%	86.7%	62.8%	85.5%
Purchase year ^4^	2015 (*n* = 318)	94.0%	90.6%	89.9%	85.9%	86.2%	87.4%	60.1%	84.9%
	2016 (*n* = 1369)	95.8%	93.4%	92.6%	91.8%	91.8%	92.3%	68.0%	89.4%
	2017 (*n* = 1030)	96.9%	94.4%	92.5%	93.1%	91.8%	91.0%	64.4%	89.2%
	2018 (*n* = 1030)	96.2%	94.8%	93.9%	92.6%	92.0%	93.6%	57.4%	88.6%
Weekly mortality	0%	100.0%	100.0%	100.0%	100.0%	100.0%	100.0%	100.0%	100.0%
	0% to ≤2%	2.9%	0.6%	7.4%	5.2%	5.8%	2.7%	95.0%	17.1%
	2% to <5%	0.0%	0.0%	0.0%	0.0%	0.0%	0.0%	38.8%	5.5%
	≥5%	0.0%	0.0%	0.0%	0.0%	0.0%	0.0%	0.1%	0.0%

^1^ Unweaned and mixed purchase groups. ^2^ Average purchase weight (kg). ^3^ Head is the number of cattle in the purchase group at time of purchase. ^4^ The year 2019 was omitted from table due to small sample size (*n* = 2 purchase groups).

**Table 7:** Sensitivity and specificity ^1^ of detecting low mortality (0% to 0.25% mortality or 0% to 0.50% mortality) purchase groups of feedlot cattle in weeks after arrival at a feeding location (*n* = 4250 purchase groups) ^2^.

	Ability to Detect Purchase Groups with 0–0.25% Mortality						
	Week 1	Week 2	Week 3	Week 4	Week 5	Week 6	Day 43 to slaughter
Sensitivity	98.3%	83.2%	69.1%	69.6%	71.6%	82.8%	0.0%
Specificity	9.8%	33.6%	51.7%	51.2%	56.8%	30.1%	100.0%
	**Ability to Detect Purchase Groups with 0** **–0.50% Mortality**						
	Week 1	Week 2	Week 3	Week 4	Week 5	Week 6	Day 43 to slaughter
Sensitivity	98.3%	83.2%	69.2%	69.7%	71.7%	82.8%	0.0%
Specificity	10.1%	34.5%	53.6%	53.0%	58.7%	30.6%	100.0%

^1^ Sensitivity was calculated as the percent of low mortality (0% to 0.25% mortality or 0% to 0.50% mortality) in purchase groups that were accurately predicted to have low mortality. Specificity was calculated as the percent of purchase groups that did not have low mortality that was accurately identified as not having low mortality. ^2^ Additional details of the models are presented in Table 4, Table 5 and Table 6.

## Data Availability

The raw data are not publicly available to ensure confidentiality. However, any data summaries can be made available on request.

## Supplementary Materials

The following supporting information can be downloaded at: https://www.mdpi.com/article/10.3390/pathogens11040473/s1, Table S1: Description of predictors considered in random forest models predicting all-cause mortality among purchase groups of feedlot cattle.
