# Supplementary material for: Predictive Models for Weekly Cattle Mortality after Arrival at a Feeding Location Using Records, Weather, and Transport Data at Time of Purchase
Source: Pathogens. 2022 Apr 15;11(4):473. doi: 10.3390/pathogens11040473 (PMC9024862; doi:10.3390/pathogens11040473)
Supplement: Supplementary file 1 [file pathogens-11-00473-s001.zip › pathogens-1629994-supplementary.pdf]

Supplementary Table S1: Description of predictors considered in random forest models predicting all-cause mortality among purchase groups of feedlot cattle

Predictor	Description	Coding
Age	Age of purchase group	1 = Calf and mixed, 2 = Yearling
Weaned status	Weaned status of purchase group	1 = Mixed and unweaned, 2 = Weaned
Sex	Sex of purchase group	1 = Female, 2 = Male
Origin	Geographic region where purchased	1= Canada or north US, 2= South or mixed
Nationality	Country where purchased	1 = Canada, 2 = US
Average purchase weight	Average purchase weight of purchase group	Continuous
Month	Month purchased	1 = January, 2 = February, … 12 = December
Day of week	Day of week purchased	1= Monday, 2= Tuesday,… 6 = Saturday/Sunday
Week of month	Week of month purchased	1 = Days 1-7, 2 = Days 8-14, 3 = 15-21, 4 = Day 22 until end of month
Season	Season when purchased	1= Spring (March, April, May), 2 = Summer (June, July, August), 3 = Fall (September, October, November), 4= Winter (December, January, February)
State	State where purchased, states with small sample size grouped together based on location	1 = CO, 2 = FL, GA, AL, MS, SC, 3 = ID, NV, OR, UT, CA, WY, MT, 4 = IL, OH, WI, 5 = IA, 6= KS, 7 = KY, 8 = MN, 9 = MS, AR, 10 = NE, 11 = ND, 12 = OK, TX, 13 = SD, 14 = TN, 15 = N/A (Canada)
Source	Source where purchased	1= Auction and mixed, 2=contracted
Shipping distance	Distance from purchase location to backgrounding/feedlot location	Continuous
Head	Number of cattle in purchase group	Continuous
Relative humidity	Humidity on purchase day	Continuous
Windspeed	Windspeed on purchase day	Continuous
Wind gust	Speed of fastest wind gust on purchase day	Continuous
Wind bearing	The direction that the wind is coming from in degrees on purchase day	Continuous
Maximum temperature	Maximum temperature on purchase day	Continuous
Minimum temperature	Maximum temperature on purchase day	Continuous
Minimum apparent temperature	Minimum "feels like" temperature on purchase day	Continuous
Maximum apparent temperature	Maximum "feels like" temperature on purchase day	Continuous
Maximum precipitation intensity	Maximum precipitation intensity on purchase day	Continuous
Precipitation intensity	The intensity (water per hour) of precipitation	Continuous
Precipitation	Precipitation on purchase day	0 = There was not precipitation, 1= There was precipitation
Precipitation type	Type of precipitation on purchase day	1 = Rain, 2 = Snow, 3 = Sleet
Precipitation accumulation	The amount of snowfall accumulation expected to occur in inches on purchase day	Continuous
Ozone	The columnar density of total atmospheric ozone on purchase day	Continuous
Visibility	The average visibility in distance on purchase day	Continuous

Weather icon	Text icon of weather on purchase day	1 = Clear day, 2 = Cloudy, 3 = Fog, 4 = Partly cloudy day, 5= Rain, 6 = Sleet, 7 = Snow, 8 = Wind
Dew point	The atmospheric temperature below which water droplets begin to condense on purchase day	Continuous
Pressure	The sea-level air pressure on purchase day	Continuous
UV index	UV radiation levels on purchase day	Discrete scale 0-11
